# Evaluation of the Genetic Diversity and Population Structure of Four Native Pig Populations in Gansu Province

**DOI:** 10.3390/ijms242417154

**Published:** 2023-12-05

**Authors:** Zunqiang Yan, Kelin Song, Pengfei Wang, Shuangbao Gun, Xi Long

**Affiliations:** 1 College of Animal Science and Technology, Gansu Agricultural University, Lanzhou 730070, China; yanzq@gsau.edu.cn (Z.Y.); 17899312494@163.com (K.S.); wangpf815@163.com (P.W.); 2 Chongqing Academy of Animal Sciences, Chongqing 402460, China

**Keywords:** single nucleotide polymorphisms chip, genetic diversity, population structure, conservation, indigenous pig

## Abstract

Indigenous pig populations, including Bamei pigs (BM), Hezuo pigs (HZ), Huixian Qingni Black pigs (HX), and Minxian Black pigs (MX) in Gansu Province, live in a particular climate and a relatively closed geographical environment. These local pig breeds are characterized by excellent characteristics (e.g., cold tolerance, robust disease resistance, and superior meat quality). In the past few years, pig populations in Gansu Province have decreased significantly because of their poor lean meat percentage, high fat content, and slow growth rate. Maintaining the diversity of these four breeds can act as a source of new alleles to be incorporated into commercial breeds which are more susceptible to disease and less adaptable to changing conditions because of inbreeding. Genomic data analysis is adequate for determining the genetic diversity and livestock breeding population structure, even in local pig populations. However, the genetic diversity and population structure of the four native pig populations in Gansu Province are still unknown. Thus, we used “Zhongxin-I” porcine chip for the SNP detection of 102 individuals living on four pig conservation farms. A total of 57,466 SNPs were identified among the four pig breeds. The linkage disequilibrium (LD) plot showed that MX had the highest level of LD, followed by BM, HZ, and HX. The observed heterozygosity (Ho) in all four populations was higher than the expected heterozygosity (He). A principal component analysis (PCA) demonstrated that the four local pig populations were isolated. The identity displayed by the state matrix and G matrix heat map results indicated that small numbers of individuals among the four pig breeds had a high genetic distance and weak genetic relationships. The results of the population genetic structure of BM, HZ, HX, and MX pigs showed a slight genetic diversity loss. Our findings enabled us to better understand the genome characteristics of these four indigenous pig populations, which will provide novel insights for the future germplasm conservation and utilization of these indigenous pig populations.

## 1. Introduction

Domesticated pigs (*Sus scrofa*), a species originating from wild boar, have played indispensable roles in productive human life [1,2]. Chinese farmers have contributed to forming various pig breeds (e.g., Taihu pig with a high litter size and Tibetan pig with strong adaptability) in different climate environments (e.g., tropical climates, cold climates, and low-oxygen climates). This long history of pig breeding in China has led the country to bear the largest number of pig breeders worldwide [3,4,5]. The data show that China has a rich variety of pig breeds, reaching 102 breeds (including 83 local breeds, 13 cultivated breeds, and six introduced breeds), with local breeds accounting for 81.37% of the total in China. Local pig breeds in China can be classified into six major types based on their geographical distribution, namely the North China type, South China type, Central China type, Southwest China type, Plateau type, and Jianghai type, which exhibit excellent characteristics (e.g., good meat quality, robust disease resistance, and strong vitality) [6,7,8]. Recently, market forces have been pushing out local breeds all over the world, because local pig breeds often have long growth cycles and poor lean meat percentages [6]. China is now a major pig-raising nation due to it having the highest production and inventory of pigs worldwide. Many pig farms are more likely to produce European pig breeds (e.g., Landrace, Yorkshire, Duroc, Pietran, and Hampshire) because of their high lean meat production, reasonable growth rate, and excellent feed conversion efficiency [9]. These commercial pigs have gradually become dominant in the Chinese pig industry [4]. As a result, many local pig breeds have been replaced or crossed with imported pig breeds, leading to the mixing of bloodlines or even the extinction of some valuable breeds [10,11]. Livestock genetic resources are national strategic resources related to seedstock industry revitalization. Due to their essential role in agriculture in China, pig genetic resources must be taken seriously [12]. Strengthening the conservation of pig genetic resources is essential, because conserving the diversity of indigenous pig breeds has cultural value and conserves candidate genes for disease resistance for commercial breeds’ genetic improvement. It is thus important for the sustainable development of Chinese animal husbandry [13,14].

Gansu Province, located in northwest China, has a unique climate characterized by cold temperatures and less rainfall. The special climatic conditions and the relatively closed geographical environment have generated four well adapted pig breeds: Bamei (BM), Hezuo (HZ), Huixian Qingni (HX), and Minxian (MX). These germplasm resources display many attractive traits (e.g., stress resistance, strong disease resistance, and high-quality meat). The Hezuo pig is also a plateau miniature pig commonly treated as a model organism for investigating plateau adaptability [15,16].

There is no denying that commercial pig breeds threaten indigenous pigs due to their excellent performance [1]. Commercial pigs have resulted in an apparent sharp decline in the indigenous pig breed population in Gansu Province. The Chinese government has taken many measures and efforts to conserve valuable indigenous pig breeds, such as establishing provincial conservation farms and performing national conservation programs. Additionally, the population structure of these four indigenous pig breeds in Gansu Province suffered considerable reductions due to African swine fevers outbreaks. However, the genetic structure and diversity of these four pig breeds based on genome level remain unknown. Thus, it is necessary to thoroughly investigate the genetic structure and diversity of existing pig breeds in Gansu Province. This contributes to managing effective conservation measures for farmers and maintaining the diversity of these pig breeds. In this paper, based on the data of the “Zhongxin-I” porcine chip, we selected BM pigs, HZ pigs, HX pigs, and MX pigs from conserved populations as the research objects to conduct a comprehensive analysis of the phylogenetic relationships and genetic diversity among four pig breeds in Gansu Province. Our findings will provide new insights into scientific conservation and management strategies for these valuable genetic resources in Gansu Province.

## 2. Results

### 2.1. Genetic Variation and SNP Sites Discovery

**Figure 1:**
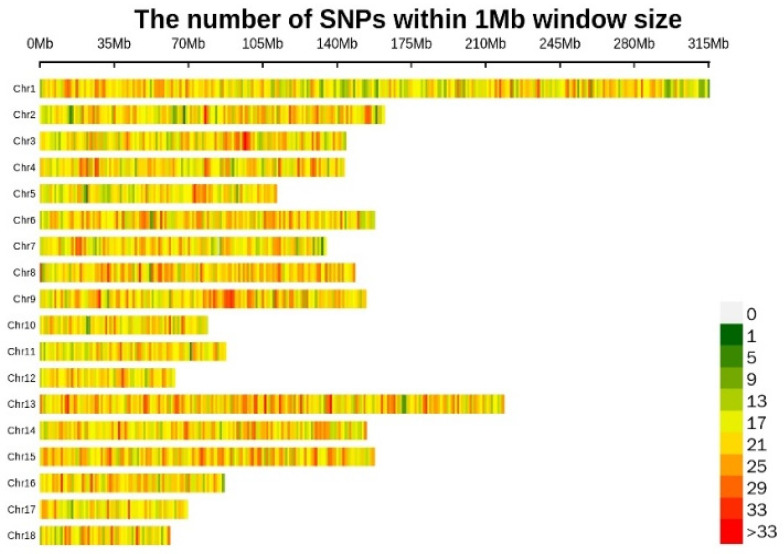
Distribution of SNPs on chromosomes.

One hundred and two samples were initially collected for genotyping with the “Zhongxin-I” porcine chip. All samples passed the quality control filter. A total of 57,466 SNPs were detected across four pig populations. Among these SNPs, there were 529 markers with a minor allele frequency (MAF) of less than 0.01. There were 721 markers with an SNP detection rate of less than 0.90. A total of 4252 markers located on the X chromosome were found. In addition, the number of SNPs with insertions and deletions was six. After quality control, 49,648 markers were retained for subsequent analysis. The distribution of SNPs on the chromosomes was visualized based on the SNP distribution plot. The results show that SNPs were distributed on all chromosomes. The results indicate that chromosome 1 contained the most SNPs (Figure 1).

### 2.2. Genetic Diversity and Linkage Disequilibrium (LD) Analysis

**Figure 2:**
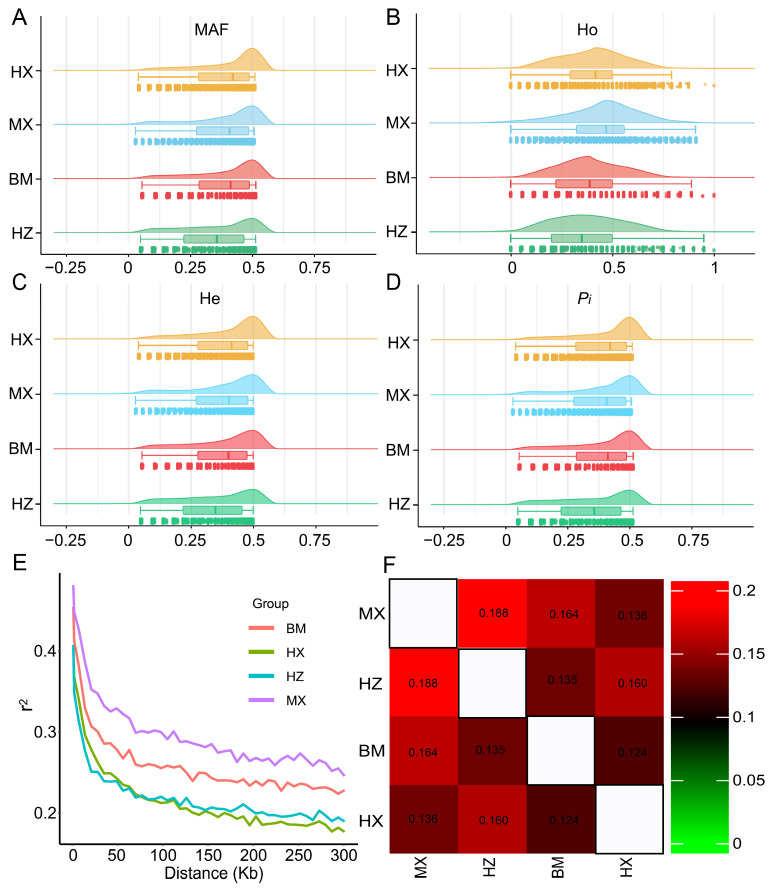
Analysis of the genetic diversity of four pig populations. Orange, blue, red and green color represent HX, MX, BM and HZ in figure (**A**–**D**), respectively. (**A**) Raincloud plot for minor allele frequency (MAF). (**B**) Observed heterozygosity (Ho) displayed by a raincloud plot. (**C**) A raincloud plot exhibiting the expected heterozygosity (He). (**D**) A raincloud plot describing the change in nucleotide diversity (*Pi*). (**E**) Drawn linkage disequilibrium (LD) decay plot describing the change in the degree of LD among the four pig populations. (**F**) Heat map of *F_ST_* distance for four pig populations.

Six statistics were calculated to evaluate the genetic diversity of the four pig breeds. We noticed that the MAF of the four indigenous pig breeds was 0.21–0.28. The MAF of the the HX population was the highest at 0.28, followed by the MX, BM, and HZ populations (Table 1, Figure 2A). Heterozygosity is often used to calculate the genetic diversity of populations. This paper used the observed heterozygosity (Ho) and expected heterozygosity (He) parameters to measure the genetic diversity of four pig populations. The Ho in all four populations was higher than He. The HZ pig population was found to have the lowest Ho and He values at 0.35 and 0.32, respectively. At the same time, the MX pig population showed the highest Ho value at 0.43. The HX pig population had the highest He value at 0.37 (Table 1, Figure 2B,C). Regarding nucleotide diversity (*P_i_*) across four indigenous pig populations, the HX pig population displayed the highest *P_i_* value at 0.39. In contrast, the HZ pig population had the lowest *P_i_* value at 0.33 (Table 1, Figure 2D). We also evaluated the polymorphic marker ratio (*P_n_*) and effective population content (Ne) based on the autosomal genome information of the HX, MX, BM, and HZ conserved pig populations. The HZ pig population had the lowest *P_n_* value at 0.82. In contrast, the HX pig population had the highest *P_n_* value at 0.92 (Table 1). The Ne values of the HX, MX, BM, and HZ pig populations were 2.2, 2.5, 1.4, and 1.7, respectively (Table 1).

Among the four populations, the LD plot showed that the MX pig population had the highest level of LD, followed by the BM, HZ, and HX pig populations. The LD coefficient (*r*^2^) decreased as the distance between loci increased in all four populations. The decay rate of LD was observed to be the fastest in the HX pig population, which is consistent with the LD plot results (Figure 2E). We used the *F_ST_* value for statistical analysis to determine the genetic dissimilarity between each pair of four pig populations. The genetic differentiation among the HX, MX, BM, and HZ pig populations ranged from 0.12 to 0.19. The genetic differentiation in MX vs. HX and HZ vs. BM was 0.14 and 0.13, respectively. BM vs. HX had the lowest genetic differentiation value, at 0.12. The highest genetic differentiation value was 0.19 in the HZ vs. MX populations (Figure 2F).

**Figure 3:**
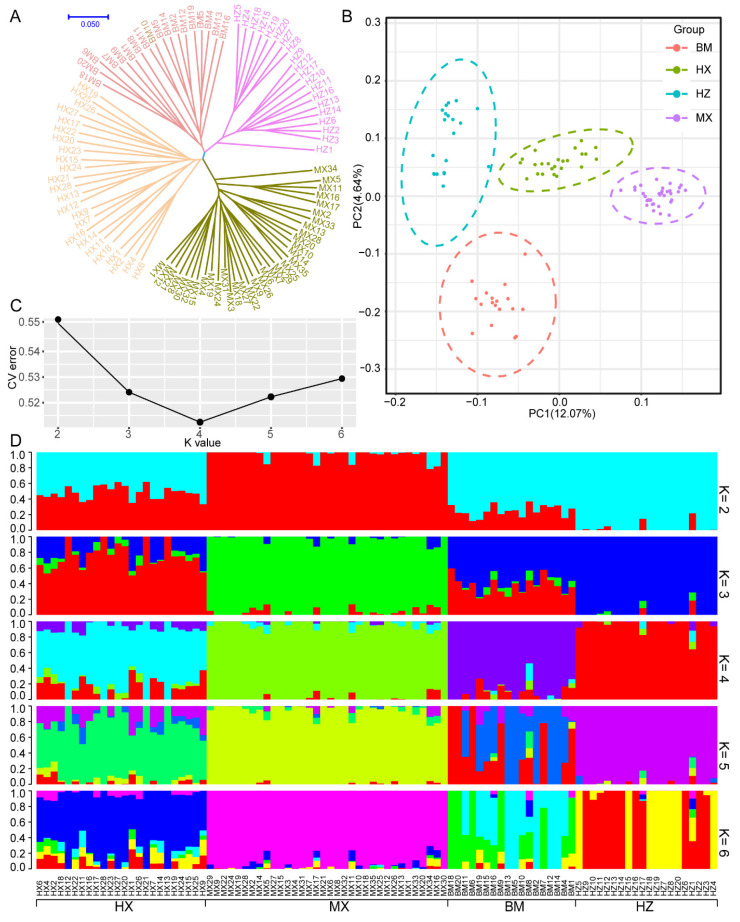
Analysis of the genetic structure of four pig populations. (**A**) A neighbor-joining tree for all individuals. (**B**) Plot of the first and second principal components resulting from a principal component analysis of all pig populations. (**C**) The cross-validation error rate. (**D**) The plot of the ancestry compositions of four breeds uncovered with the assumed number of ancestries (K = 2–6). Each column represents an individual, in which the length of different colors fragments represents the proportion of an ancestor in the individual genome. A common color represents one ancestral cluster and the abscissa represents the group name.

A neighbor-joining (NJ) tree was created to investigate the genetic distances among the four indigenous pig breeds, providing a clear phylogenetic relationship among these pigs. The NJ tree for the four pig populations showed that the HX, MX, BM, and HZ pig populations have a monophyletic clade (Figure 3A). Principal component analysis (PCA) can identify the principal components representing the population structure based on individual genetic correlations. The PCA analysis displayed four local pig populations, indicating significant genetic differences between these groups. The first and second PCA explained 12.07% and 4.64% of the total variation, respectively (Figure 3B). We observed two to six subgroups (K-values) based on the research population and performed cross-validation on the clustering results. Based on the valley value of the cross-validation error rate, we determined that the optimal number of subgroups was K = 4 (Figure 3C). According to the population structure analysis, four populations were distinguished from each other when K was equal to 4 (Figure 3D).

### 2.3. Genetic Distance of the Conserved Population

**Figure 4:**
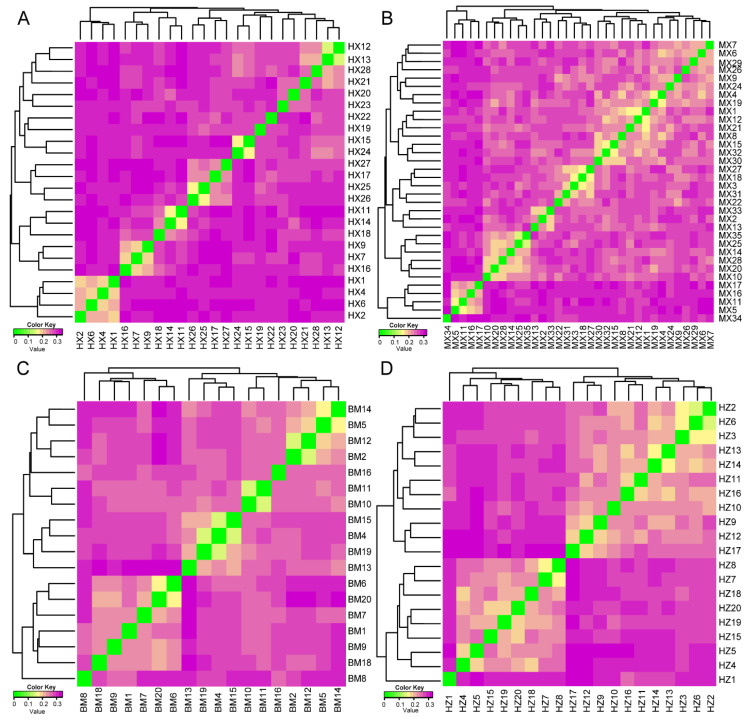
Identity by state (IBS) distance matrix of HX (**A**), MX (**B**), BM (**C**), and HZ (**D**) pig-conserved populations. Each small square in the figure represents the genetic distance value between the two pairs from the first individual to the last individual. The smaller the value (the closer it is to green), the smaller the genetic distance between two individuals, meaning they were extremely similar, and vice versa.

Based on the identity by state (IBS) genetic distance matrix of the population structure, among the four populations, we found that small numbers of individuals had high genetic distances (Figure 4). Across the four indigenous pig populations, the HX pig population had the highest average genetic distance value at 0.29, ranging from 0.14 to 0.34 (Figure 4A). The MX pig population had the lowest average genetic distance value at 0.26, ranging from 0.14 to 0.32 (Figure 4B). We calculated the IBS distance as varying from 0.16 to 0.37 for the conserved population of BM pigs (Figure 4C). The genetic distance ranged from 0.15 to 0.33 in the HZ conserved pig population (Figure 4D).

**Figure 5:**
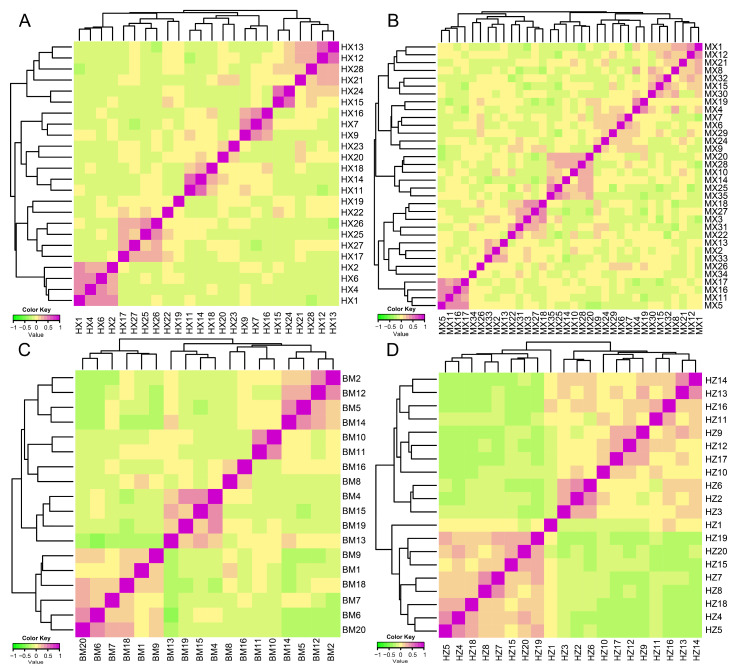
**The** G matrix heat map of the conserved population of HX (**A**), MX (**B**), BM (**C**), and HZ (**D**) pigs. Each small square exhibits the value of the relationship between two pairs, from the first individual to the last individual. The larger the value (the closer it is to purple), the closer the relationship between two individuals.

We also calculated the genetic relationship based on the G matrix. The visualization results of the G matrix of the four conserved populations showed that most individuals have moderate genetic relationships (Figure 5). We found that the genetic relationship of the MX population was the weakest, with a value of 0.03 (Figure 5B). Additionally, the HZ pig population produced the highest value for its genetic relationship at 0.05 (Figure 5D). The genetic relationship values of the BM and HX pig populations were 0.04 and 0.04, respectively (Figure 5A,C).

## 3. Discussion

Domestic animal (including livestock and poultry) genetic diversity is closely related to human production, representing valuable genetic resources for cultivating new and excellent breeds of domestic animals and improving the core competitiveness of livestock products [17]. Human beings must protect domestic animal genetic diversity, because livestock genetic diversity can drive the high-quality development of animal husbandry, accelerate the progress of human civilization and promote economic development [18,19,20]. Based on data from the Domestic Animal Diversity Information System and Food and Agricultural Organization in 2004, one-third of the pig breeds in the world are cultivated in China [2,20,21]. However, some commercial breeds account for the highest proportion of genetics utilized for production and dominate the Chinese pig industry. Consequently, the number of Chinese indigenous breeds has decreased considerably over the past few decades [18].

The HX, MX, BM, and HZ pig breeds, which are essential Chinese genetic resources, are mainly distributed in the Gansu Province of China. These pigs are distinguished by their moderate intramuscular fat content, good flavor, tender meat quality, apparent cold tolerance and robust disease resistance. Western commercial pigs threaten to replace these pig breeds, leading to the erosion of valuable and particular genetically adapted resources in Gansu Province. Genetic diversity and population structure analyses are crucial for protecting these indigenous pig populations [21]. This study aimed to determine the population structure and genetic diversity to provide rational scientific conservation strategies for HX, MX, BM, and HZ pig populations. More and more studies have investigated the genetic diversity and population structure of various species of livestock (e.g., Min pig, Tongcheng pig, Tibetan sheep, Hainan cattle, and BoHuai goat) using whole-genome sequencing or SNP chips [14,22,23,24,25,26,27]. For instance, Meng et al. used a KPS porcine breeding chip v1 50K for SNP to study the genetic diversity of the Min pig-conserved population. They found that the genetic diversity of the conserved Min pig population was low, indicating a certain degree of inbreeding in the Min pig [28]. Yang et al. performed whole-genome sequencing to detect the population genetic structure and diversity of Beijing Black pigs. They demonstrated that the core population size was small in the conserved farm. However, existing breeding schemes effectively avoid inbreeding to a certain extent [11]. These studies indicated that using whole-genome sequencing or SNP chips to explore genetic diversity and population structure provides the necessary data to implement conservation programs.

This study used high-density SNP chip data to evaluate the conservation state of HX, MX, BM, and HZ pig populations for the first time. The expected heterozygosity (He) values of HX, MX, BM, and HZ conserved pig populations were lower than those in Jinhua pigs (He = 0.43) [29], Laiwu pigs (He = 0.38) [30], Erhualian pigs (He = 0.38) [5], and Meishan pigs (He = 0.38) [5]. The He values of HX, MX, BM, and HZ conserved pig populations were higher than those of Min pigs (He = 0.33) [28], Liangshan pigs (He = 0.35) [20], Wuzhishan pigs (He = 0.31) [31], and Licha Black pigs (He = 0.35) [12]. The moderate He of HX, MX, BM, and HZ conserved pig populations demonstrated that many SNP loci have been subjected to inbreeding. Additionally, the observed heterozygosity (Ho) values were higher than those of He in this study, indicating that they have been influenced by gene flow (such as the introduction of a foreign lineage). Unlike Western commercial pigs, indigenous pigs in China do not undergo intense artificial selection pressure [31]. As a consequence, Chinese indigenous pigs display abundant genetic diversity. The values of the *P_i_* parameter of the four pig populations in this study were 0.38, 0.36, 0.37, and 0.33, respectively, which were higher than those of commercial pig breeds, such as Landrace pig (*P_i_* = 0.0019) [31] and Large White pig (*P_i_* = 0.0018) [31]. Effective population size (Ne) is an important parameter in pig genetic breeding. We speculated that some excellent commercial pig breeds have been introduced, leading to threats to indigenous pig breeds in Gansu Province. Although the conserved population of the four pig breeds were established, the Ne remained low because of the limited number of unique families available. It is a fact that many indigenous domestic animals in China are facing the same situation. Thus, it is vital to immediately minimize consanguinity in indigenous pig breed populations by paying more attention to breeding plans.

LD describes the nonrandom linear association of alleles at different loci generated by migration, selection, and genetic drift [32]. LD analysis can reveal the intensity of selection that breeds have been subjected to during their evolutionary process and provide information on the evolution of the population [33,34]. When selection drives LD, the slower the LD decay, the greater the intensity of selection, thereby inferring the differences in the selection intensity in various populations. This study found that the LD coefficients of the MX pig populations were highest among the four populations, followed by BM, HZ, and HX. These results suggest that the four pig populations posed weaker artificial selection intensity during domestication. Herein, we analyzed the genetic structure among four pig populations using a phylogenetic tree, PCA, and structure methods. Considering the significance of the pig population in the breeding process, the phylogenetic tree of pig populations was drawn using the NJ method. The phylogenetic tree has monophyletic clades for each breed, including BM, HZ, HX and MX. When K = 4 in the structure analysis, a clear division among the four pigs was observed, consistent with the *F_ST_* value calculated between each other. These results suggest that the four local pig breeds displayed obvious genetic differences because of their unique geographic isolation.

Currently, most of the local pig breeds in China are conserved by closed breeding, which leads to the genomic kinship of the population being easily affected by selection. Therefore, understanding the kinship of the conserved populations is crucial for conserving the populations. The genomic kinship matrix established using a whole-genome SNP marker panel reflects the individual kinship when the marker density is suitable. In this study, IBS distance matrix and G matrix analyses showed that most individuals in the HX, MX, BM, and HZ pig populations were distinct, and few individuals were close to each other. Meanwhile, we calculated the genetic distance among the four pig populations as 0.29, 0.26, 0.29, and 0.26, respectively, which is approximately equal to the genetic distance of Liangshan pigs (0.28) [20].

## 4. Materials and Methods

### 4.1. Animal Care

All animal work was conducted following the guidelines on the care and use of experimental animals established by the Ministry of Agriculture in China. The Committee on Animal Ethics of Gansu Agricultural University approved this study under Permit No. 2006-398.

### 4.2. Animal and Sample Collection

One hundred and two ear tissue samples, including 20 BM pigs, 20 HZ pigs, 28 HX pigs, and 34 MX pigs, were randomly collected from conservation farms and core areas of origin (Table 2).

### 4.3. DNA Extraction

Genomic DNA was extracted from the ear tissues using a standard phenol/chloroform extraction method. DNA integrity and purity were detected using a Nanodrop 2000 nucleic acid protein analyzer (Thermo, Shanghai, China) and agarose gel electrophoresis (BIO RAD, Wuhan, China). The extracted DNA samples were stored at −20 °C for further analysis.

### 4.4. Single Nucleotide Polymorphism Genotyping

The qualified 102 DNA samples were genotyped with the “Zhongxin-I” porcine chip containing 51,315 SNPs (Beijing Compass Biotechnology Co., Ltd., Beijing, China), which provided the highest throughput array format based on the data. Nine populations of Chinese local pigs (e.g., Bamaxiang pig, Sutai pig, and Laiwu pig) provided reference genome information [28]. Therefore, the “Zhongxin-I” porcine chip was selected for this study.

### 4.5. Quality Control

Strict quality control criteria were implemented to enhance the accuracy of the data processing. The genotype data underwent quality evaluation using Plink (v1.90) software [35]. The panel eliminated unreliable genotypes that did not meet the following four criteria [28]: (i) individuals with an SNP call rate greater than 90%; (ii) SNP MAF greater than 0.01; (iii) Hardy–Weinberg equilibrium (HWE) test *p*-value less than 0.000001; and (iv) SNPs of autosomal loci were used.

### 4.6. Population Genetic Diversity Analysis

Plink (v1.90) software [35] was employed to compute the expected heterozygosity (He), observed heterozygosity (Ho), and polymorphic loci ratio (*P_n_*). Nucleotide diversity (*P_i_*) and the population differentiation index (*F_ST_*) were calculated using VCFtools (v0.1.17) software [36]. Additionally, population effective size (Ne) was determined using SNeP (v1.1) software [37] to analyze the genetic diversity of the BM, HZ, HX, and MX pig populations in Gansu Province. Additionally, raincloud plots for the MAF, Ho, He, and *P_i_* statistical indicators in the four pig populations were obtained using OmicShare tools, a free online platform for data analyses (http://www.omicshare.com/tools (accessed on 6 October 2023)).

### 4.7. Population Genetic Structure Analysis

PCA of the four pig populations was performed using GCTA (v1.94) software [38]. An evolutionary tree of the four pig populations was constructed using MEGA (v11) software [39], employing the NJ method. Admixture analysis was performed at various levels (K = 2 to 6) for the four populations using Admixture (v1.30) software [40].

### 4.8. LD Analysis

LD refers to the nonrandom combination of alleles at different population loci [41]. It indicates that the probability of the simultaneous occurrence of two alleles on the same chromosome is higher than the probability expected based on random distribution within the population [42,43]. This condition is called LD and is commonly represented using D’ and *r*^2^ values. This study analyzed the LD levels of four pig populations using PopLDdecay (v1.90) software [44].

### 4.9. Population Differentiation Index Analysis

*F_ST_*, the population genetic differentiation index, measures population differentiation and genetic distance. Its values range from 0 to 1. A value of 0 indicates random mating and complete genetic similarity between two populations, while 1 implies complete isolation and no genetic similarity between two populations. *F_ST_* analysis was performed using VCFtools (v0.1.17) software [36] in this study.

### 4.10. Relatedness Analysis

Plink (v1.90) software [35] was used to calculate the genetic distance based on identity by state (IBS) for the four pig populations, and an IBS matrix was constructed. This matrix was then used to analyze the genetic distance between individuals within the population. GCTA (v1.94) software [38] was utilized to build the G matrix, which was used to analyze relatedness among individuals within the population of four pig populations. The results of the analysis were visualized using R programming language.

## 5. Conclusions

This study analyzed the genetic diversity and structure of HX, MX, BM, and HZ pig populations at the molecular level based on 50K SNP chip data. We found that the genetic diversity and the effective content of the conserved HX and MX populations were high. These results demonstrate that conserved pig populations were unstratified purebred populations with rich genetic diversity, indicating that the HX and MX pig populations have been effectively conserved. However, the genetic diversity level of the BM and HZ pig populations was inadequate. Thus, we need to take some effective measures to avoid inbreeding to ensure the long-term preservation of genetic resources via establishing preservation measures and formulating a scientific mating plan. This study will provide valuable information for the conservation, protection, and utilization of Gansu-native pigs in China.

## Figures and Tables

**Table 1:** Genetic diversity indicators of four indigenous pig breeds in Gansu Province.

Breed	MAF	Ho	He	*P_i_*	*Pn*	Ne
HX	0.28	0.39	0.37	0.38	0.92	2.2
MX	0.24	0.43	0.36	0.36	0.82	2.5
BM	0.24	0.37	0.36	0.37	0.85	1.4
HZ	0.21	0.35	0.32	0.33	0.82	1.7

**Table 2:** Basic information on samples from four domestic pig breeds in Gansu Province.

Breed	Abbreviation	Sample Type	Sample Size	Sampling Location
Bamei pig	BM	Ear tissue	20	Lingtai, Pingliang, Gansu
Hezuo pig	HZ	Ear tissue	20	Hezuo, Gannan, Gansu
Huixian Qingni Black pig	HX	Ear tissue	28	Huixian, Longnan, Gansu
Minxian Black pig	MX	Ear tissue	34	Minxian, Dingxi, Gansu

## Data Availability

The raw data used in this paper are publicly available and can be obtained upon reasonable request from the corresponding author.
